# Impact of Antibiotics on the Subgingival Microbiome in Advanced Periodontitis: Secondary Analysis of a Randomized Controlled Trial

**DOI:** 10.3390/diagnostics15162012

**Published:** 2025-08-11

**Authors:** Behrouz Arefnia, Ingeborg Klymiuk, Stefanie Anna Peikert, Jakob Sebastian Bernhard, Gerald Seinost, Gernot Wimmer

**Affiliations:** 1 Division of Restorative Dentistry, Periodontology and Prosthodontics, Department of Dental Medicine and Oral Health, Medical University of Graz, 8010 Graz, Austria; 2 Division of Cell Biology, Histology and Embryology, Gottfried Schatz Research Center, Medical University of Graz, 8010 Graz, Austria; 3 Division of Angiology, Department of Internal Medicine, Medical University of Graz, 8010 Graz, Austria

**Keywords:** adjunctive systemic antibiotics, next-generation sequencing, one-stage full-mouth disinfection, severe periodontitis, subgingival microbiome

## Abstract

**Background/Objectives**: This study aimed to evaluate longitudinal changes in the subgingival microbiome over 12 months following non-surgical periodontal treatment, with or without adjunctive systemic antibiotics, in patients with stage III/IV periodontitis and peripheral artery disease. **Materials**: After randomizing patients to full-mouth mechanical debridement with/without adjunctive systemic antibiotics (PT1/PT2 group) or no subgingival debridement (control group), periodontal probing depths were measured, scores for ‘periodontal inflamed surface area’ (PISA) obtained, and subgingival plaque samples collected at baseline and during the 3-month and 12-month follow-up visits. Next-generation 16S DNA sequencing was used to characterize the microbiota of the samples for alpha/beta diversity and differentially abundant taxa. **Results**: Complete data was available for 76 patients. At 3 months, shallow (≤3.4 mm) or advanced (≥5.5 mm) pockets were significantly more, or less, prevalent in the PT1 than in the control group (*p* = 0.013/0.004). Microbiologically, the PT1 group was even more distinct, being associated with statistically significant changes over time (in alpha/beta diversity and differential taxa abundances) not seen in the PT2 and control groups. **Conclusions**: Although non-surgical treatment can reduce periodontal inflammation with or without antibiotics, subgingival microbial diversity can only be sustainably affected, and periodontitis-associated microbiota reduced, in the presence of adjunctive systemic antibiotics.

## 1. Introduction

The oral cavity represents one of the most diverse microbial ecosystems in the human body, comprising a multitude of ecological niches colonized by a vast array of microbial species that form highly organized biofilms [1]. The term microbiome encompasses the collective genomes of all commensal, symbiotic, and pathogenic microorganisms residing in or on the human body. The Human Oral Microbiome Database (eHOMD) currently catalogs 774 microbial species inhabiting the oral and non-oral aerodigestive tract, with 58% of these being formally named, 16% cultivated but unnamed, and 26% uncultivated phylotypes [2]. This microbial complexity plays a central role in the etiology and progression of periodontal diseases. Periodontitis arises from multifactorial interactions between dysbiotic microbial communities, host susceptibility, immune responses, and environmental factors such as smoking and diet [3]. Inflammatory responses driven by an ecological shift toward specific pathogenic consortia are considered causative in disease progression [4,5]. While traditional culture methods have been limited in capturing this diversity, and early molecular diagnostics focused on a narrow range of pathogens, recent advances in next-generation sequencing (NGS), bioinformatics, and statistical modeling have significantly improved our ability to characterize the subgingival microbiome [6,7,8,9]. Despite these advancements, relatively few studies have explored how periodontal therapy—particularly with or without adjunctive systemic antibiotics—alters the subgingival microbial composition over time [10,11,12]. A recent long-term analysis demonstrated that adjunctive systemic antibiotics can sustainably reduce subgingival dysbiosis up to 26 months after therapy, despite only transient changes in microbial diversity. These findings underscore the potential of antibiotic-supported therapy to induce ecologically stable, health-associated shifts in the subgingival microbiome [13]. Clinical evidence supports the effectiveness of mechanical biofilm debridement [14], and systematic reviews and meta-analyses suggest added clinical benefit from systemic antibiotics in selected patient populations, particularly those with severe or active disease [15,16]. This study aimed to compare clinical and microbiological outcomes over a 12-month period in patients with stage III/IV periodontitis and peripheral artery disease undergoing full-mouth non-surgical periodontal therapy with or without adjunctive systemic antibiotics, relative to a control group. Peripheral artery disease (PAD) and periodontitis are both chronic inflammatory conditions that share common risk factors and pathophysiological mechanisms, including systemic inflammation and endothelial dysfunction [17,18]. Investigating patients with PAD thus offers a clinically relevant model to explore how periodontal therapy may influence systemic health and subgingival microbiome dynamics in a high-risk population.

We hypothesized that systemic antibiotic therapy would result in more pronounced and sustained shifts in subgingival microbial diversity and composition than mechanical therapy alone.

## 2. Materials and Methods

This investigation constitutes a secondary analysis of a previously registered randomized controlled trial (RCT; German Clinical Trials Register: DRKS00004554). While the original study focused on short-term clinical outcomes at the 3-month follow-up [19,20], the present analysis extends the observation period to 12 months and places a novel focus on longitudinal microbiome dynamics in conjunction with clinical markers such as periodontal inflamed surface area (PISA). This secondary analysis also includes an expanded microbiological sample set and places particular emphasis on differences between groups in subgingival microbial diversity and composition.

The study was conducted in accordance with the Declaration of Helsinki. Ethical approval was granted by the Medical University of Graz ethics committee (reference number 24-456 ex 11/12). All participants provided written informed consent prior to clinical or microbiological assessment.

### 2.1. Study Population and Design

Patients were recruited at the Division of Angiology, Department of Internal Medicine, Medical University of Graz. Key inclusion criteria were a diagnosis of stage III or IV periodontitis according to the 2018 classification [21] and the presence of peripheral artery disease (Rutherford stages 2–4) [22]. Further dental inclusion criteria included ≥12 natural teeth (including third molars), ≥3 teeth with pocket probing depth (PPD) ≥ 6 mm, ≥2 with clinical attachment loss ≥ 5 mm, and bleeding on probing at >20% of sites. A complete list of inclusion and exclusion criteria is provided in the Supplementary File S1. A total of 90 patients were randomly assigned (via www.randomizer.at) into three groups: PT1 (Debridement + Antibiotics): Full-mouth non-surgical periodontal therapy (OSFMD) plus 7-day systemic antibiotics. PT2 (Debridement Only): Full-mouth non-surgical periodontal therapy without antibiotics. Control: No subgingival instrumentation for 3 months. All clinical procedures were performed by a single experienced clinician. As reported previously [20], 5 patients dropped out shortly after enrollment, resulting in a final cohort of 85 patients.

The control group underwent only a supragingival scaling and polishing session with oral hygiene instructions, including the Bass technique and the use of interdental devices. While no subgingival instrumentation was provided within the first 3 months, this limited intervention constituted a minimum standard of care, consistent with clinical reality where a hygiene phase typically precedes definitive periodontal therapy and can extend over several weeks. Importantly, no patient in the control group experienced complications requiring emergency treatment, and all were advised that they could seek periodontal therapy at any time. The current analysis focuses on all patients who completed the 12-month clinical examination, expanding upon the prior 3-month dataset.

### 2.2. Clinical Outcome Measures

All periodontal measurements were obtained using a pressure-calibrated Florida Probe (Florida Probe Corporation, Gainesville, FL, USA) with 0.1 mm accuracy. Probing pocket depth (PPD) measurements were categorized as shallow: ≤3.4 mm, moderate: 3.5–5.4 mm, and advanced: ≥5.5 mm. Additionally, periodontal inflamed surface area (PISA) was calculated as a quantitative marker of inflammatory burden based on the surface area of bleeding pocket epithelium [23]. PISA serves as a clinically relevant endpoint with emerging importance in periodontal risk assessment and systemic associations.

### 2.3. Subgingival Sampling

Following gentle cleaning of soft tissues with sterile cotton pellets, subgingival biofilm samples were collected using sterile Gracey curettes from the two most periodontally affected non-adjacent teeth per patient [24]. Samples were obtained prior to baseline supragingival cleaning and repeated at 3 and 12 months post-baseline at the same sites. The samples were placed in TE buffer (100 µL), frozen immediately on dry ice, and stored at −80 °C until analysis.

### 2.4. DNA Extraction and Sequencing

Total DNA was extracted as described by [25] using the MagNA Pure LC DNA Isolation Kit (Roche, Basel, Switzerland) for bacterial and fungal nucleic acids. After mechanical lysis and enzymatic digestion, purified DNA was eluted in 100 µL of buffer. The hypervariable V1–V2 region of the bacterial 16S rRNA gene was amplified using primers 27F and 357R. PCR products were pooled, normalized, and indexed, followed by gel purification and quality control using a 2100 Bioanalyzer (Agilent, Santa Clara, CA, USA). Sequencing was performed on an Illumina MiSeq (v3 600-cycle chemistry) (Illumina, San Diego, CA, USA) with a 20% PhiX spike-in.

### 2.5. Microbial Data Processing and Outcome Variables

Sequencing data were processed in QIIME v1.9.1 [26] implemented on the BioMedNode cluster (https://galaxy.medunigraz.at). Although QIIME 2 is more recent, QIIME 1 was used due to established and validated pipelines for longitudinal analysis and compatibility with legacy tools previously used by our group. Paired-end reads were merged using fastq-join [27], filtered (Phred ≥ 29, read length ≥ 70%), and decontaminated (chimera removal using Usearch v6.1 [28]). Primer sequences were trimmed with Cutadapt [29]. OTU picking was performed at 97% similarity threshold using open-reference clustering against the SILVA 128 database [30]. OTUs were taxonomically assigned, and phylogenetic trees constructed from PyNAST-aligned sequences [31]. Confidence scores for taxonomic assignments followed default SILVA criteria.

### 2.6. Statistical Analysis

Sample size was based on the original RCT’s power calculation using nQuery Advisor 4, with a required *n* = 27 per group for ANOVA (α = 0.05, power = 0.8). All clinical analyses were conducted in SPSS v29 (IBM, Armonk, NY, USA). Group differences in pocket depth categories and PISA values were evaluated via repeated-measures ANOVA with Bonferroni-corrected post hoc comparisons. Statistical significance was set at *p* ≤ 0.05. Microbiome data were analyzed using R v3.4.1. Alpha diversity metrics included Shannon Index (richness + evenness), Faith’s PD (phylogenetic diversity), and Observed OTUs (raw richness). Beta diversity was evaluated using Bray–Curtis dissimilarity and weighted UniFrac distances, visualized via principal coordinate analysis (PCoA) and hierarchical clustering. Differential abundance analyses employed LIMMA (linear models for microarray data) and random forest-based feature selection to identify discriminatory taxa.

## 3. Results

### 3.1. Nature and Size of the Patient Sample

**Figure 1:**
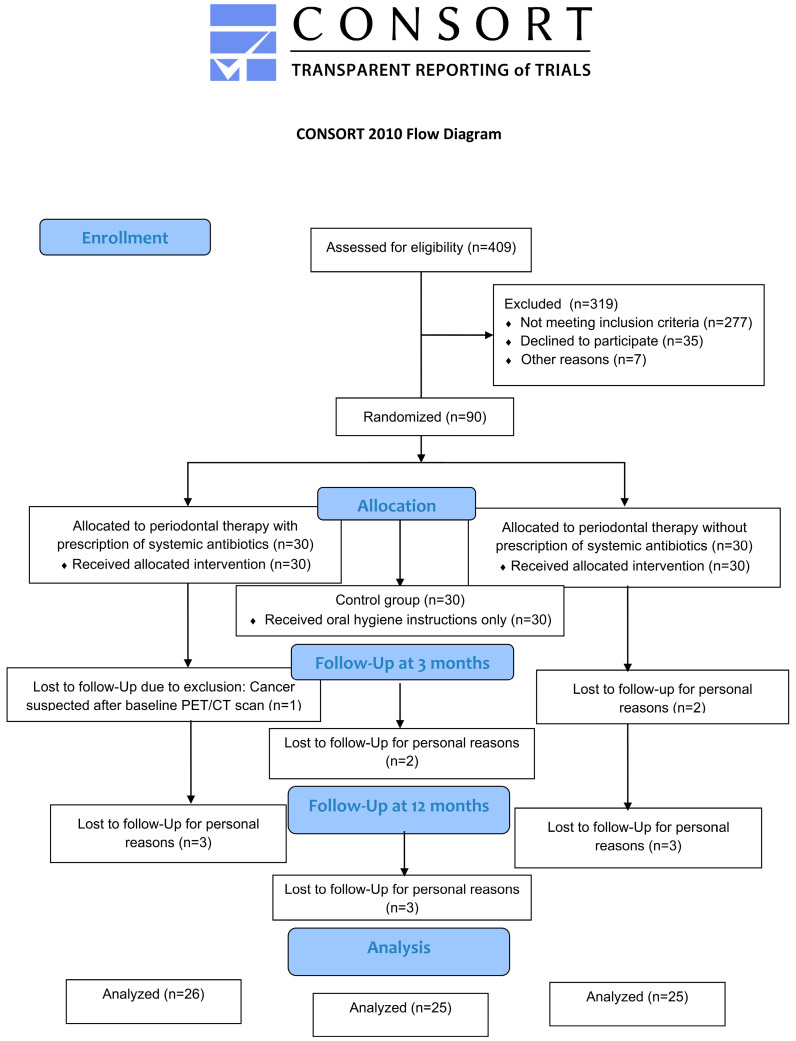
Flow chart of the study.

Clinical parameters and subgingival plaque samples could be obtained at baseline and after 3 and 12 months for 76 patients, given that 9/85 patients that had been left at 3 months were no longer willing to participate by 12 months and all 9 declined due to the long driving distance to the study site (Figure 1). Mean age at baseline was 59.9 ± 8.16 years and was comparable between the three groups (*p* = 0.835; Table 1).

**Table 1:** Demographic and clinical data about the patient cohort (*n* = 76).

	Follow-Up	PT1 Group (*n* = 26)		PT2 Group (*n* = 25)		Control Group (*n* = 25)		Test	*p*- Value
Age mean (SD)	Baseline	59.2 ± 7.72		60.6 ± 8.37		59.9 ± 8.25		†	0.835
Female, %	Baseline	13.8%		17.9%		10.7%		‡	0.712
No. of teeth mean (SD)	Baseline	21.3 ± 4.02		21.4 ± 4.96		21.6 ± 4.60		§	0.895
	3 months	20.5 ± 4.44		20.4 ± 5.25		21.6 ± 4.60		§	0.924
	12 months	20.1 ± 4.39		20.5 ± 5.43		21.2 ± 5.28		§	0.518
PISA in mm^2^ mean (SD)	Δ baseline to 3 months	−609 ± 324		−312 ± 310		−87.3 ± 200		†	<0.001
	Δ baseline to 12 months	−536 ± 376		−300 ± 379		−217 ± 382		†	0.010
	Δ 3 months to 12 months	+72.6 ± 260.1		+12.6 ± 192.7		−129.8 ± 423.8		†	0.061
Shallow pockets in % (≤3.4 mm)	Baseline	59.5%	A	61.9%	A	61.6%	A	†	0.837
	3 months	67.0%	A	64.7%	A, B	55.1%	B	†	0.013
	12 months	66.9%	A	63.8%	A	58.1%	A	†	0.102
	Δ baseline to 3 months	+7.52 ± 9.71		+2.86 ± 7.19		−6.43 ± 8.40		†	<0.001
	Δ baseline to 12 months	+6.92 ± 12.5		−0.32 ± 12.5		−6.08 ± 11.37		†	0.001
	Δ 3 months to 12 months	−0.15 ± 11.05		−3.40 ± 10.08		+1.04 ± 10.94		†	0.321
Moderate pockets in % (3.5–5.4 mm)	Baseline	31.4%	A	29.1%	A	26.1%	A	†	0.137
	3 months	27.7%	A	28.3%	A	30.6%	A	†	0.573
	12 months	27.7%	A	28.6%	A	32.3%	A	†	0.224
	Δ baseline to 3 months	−3.66 ± 8.81		−0.75 ± 5.43		+4.54 ± 7.65		†	<0.001
	Δ baseline to 12 months	−3.46 ± 9.33		+0.88 ± 8.81		+6.52 ± 7.64		†	<0.001
	Δ 3 months to 12 months	−0.19 ± 9.40		+2.08 ± 6.73		+1.20 ± 8.61		†	0.619
Advanced pockets in % (≥5.5 mm)	Baseline	9.1%	A	9.1%	A	12.4%	A	†	0.419
	3 months	5.3%	A	6.9%	A	14.3%	B	†	0.004
	12 months	5.5%	A	7.5%	A	10.1%	A	†	0.077
	Δ baseline to 3 months	−3.86 ± 5.15		−2.21 ± 4.45		+1.86 ± 2.93		†	<0.001
	Δ baseline to 12 months	−3.38 ± 6.53		−0.56 ± 7.12		−0.04 ± 9.84		†	0.275
	Δ 3 months to 12 months	+0.42 ± 4.24		+1.40 ± 5.52		−1.80 ± 9.83		†	0.252

The *p*-values in the right column refer to all three groups, any absence of the same letter (A or B) between two horizontally aligned fields indicating a significant pairwise difference (*p* ≤ 0.05). Interval-based differences are marked by a delta symbol in the “follow-up” column (Δ) and are broken down in Table 2 into post hoc pairwise comparisons. PISA: periodontal inflamed surface area (given variable tooth numbers per patient, PISA was not analyzed at specific times but in terms of interval-based changes only). PT1: non-surgical periodontal treatment with systemic antibiotics; PT2: like PT1 but without antibiotics. † ANOVA; ‡ chi-square test; § Kruskal–Wallis test.

**Table 2:** Pairwise comparisons of PISA scores and pocket depths based on intervals.

Interval	Parameter	PT1 Group vs. PT2 Group		PT2 Group vs. Control Group		PT1 Group vs. Control Group	
Δ from baseline to 3 months	PISA, mm^2^	↓↓	*p* = 0.001	↓↓	*p* = 0.018	↓↓	*p* < 0.001
	Shallow pockets, %	↑↑	n.s.	↑↓	*p* < 0.001	↑↓	*p* < 0.001
	Moderate pockets, %	↓↓	n.s.	↓↑	*p* = 0.026	↓↑	*p* < 0.001
	Advanced pockets, %	↓↓	n.s.	↓↑	*p* = 0.002	↓↑	*p* < 0.001
Δ from baseline to 12 months	PISA, mm^2^	↓↓	n.s.	↓↓	n.s.	↓↓	*p* = 0.010
	Shallow pockets, %	↑↓	n.s.	↓↓	n.s.	↑↓	*p* < 0.001
	Moderate pockets, %	↓↑	n.s.	↑↑	n.s.	↓↑	*p* < 0.001
	Advanced pockets, %	↓↓	n.s.	↓↓	n.s.	↓↓	n.s.
Δ from 3 months to 12 months	PISA, mm^2^	↑↑	n.s.	↑↓	n.s.	↑↓	n.s.
	Shallow pockets, %	↓↓	n.s.	↓↑	n.s.	↓↑	n.s.
	Moderate pockets, %	↓↑	n.s.	↑↑	n.s.	↓↑	n.s.
	Advanced pockets, %	↑↑	n.s.	↑↓	n.s.	↑↓	n.s.

Δ: interval-based difference; ↑: increase; ↓: decrease; n.s.: not significant; PISA: periodontal inflamed surface area; PT1: non-surgical periodontal treatment with systemic antibiotics; PT2: same treatment without antibiotics. All *p*-values were obtained by ANOVA post hoc testing.

### 3.2. Differential Developments in PISA Scores

From baseline to 3 months, reductions in mean PISA scores were observable in all groups (Table 1) and were significantly different between any two groups—i.e., for PT1 vs. PT2 (*p* = 0.001), controls vs. PT2 (*p* = 0.018), and controls vs. PT1 (*p* < 0.001; Table 2). At 12 months compared to baseline, the scores were still reduced in all groups (Table 1), but a significant intergroup difference was only found for PT1 vs. controls (*p* = 0.010; Table 2). The changes from 3 to 12 months were characterized both by minor increases in the PT1/PT2 groups and by reductions in the control group (Table 1) without any intergroup differences (Table 2). Notably, the sustained reduction in PISA values observed in the PT1 group over 12 months suggests a clinically relevant decrease in periodontal inflammatory burden associated with adjunctive antibiotic use. While all groups showed improvements from baseline, the magnitude of change was most pronounced and persistent in PT1, reinforcing the potential of systemic antibiotics to achieve a deeper and more stable resolution of inflammation. These findings highlight PISA as a sensitive endpoint for evaluating long-term treatment efficacy beyond standard probing metrics.

### 3.3. Depth Distributions of Subgingival Pockets

No significant intergroup differences in pocket depth were seen at baseline (Table 1). At 3 months, significant differences were noted for PT1 vs. controls based on shallow (*p* = 0.013) and for PT1/PT2 vs. controls based on advanced (*p* = 0.004) pockets. No other group comparisons at the 3- or 12-month follow-up were significant (*p* > 0.05; Table 1). No significant differential changes emerged for PT1 vs. PT2 during any intervals (Table 2). From baseline to 3 months, all changes in the control group differed significantly from those in the PT1/PT2 groups. From baseline to 12 months, they only differed from the PT1 group based on shallow and moderate pockets; and from 3 to 12 months, no significant changes were seen between any of the groups (Table 2).

### 3.4. Microbial-Community Findings

A total of 228 biofilm samples, collected at baseline and during the (3- and 12-month) follow-up visits, were available for analysis of the three study groups. Next-generation sequencing resulted in mean reads per sample of 102,802 ± 23,288 (41,070–160,654; median: 102,365). Equal sequencing depths were obtained in the three study groups, with reads per sample of 58,043 (23,589–89,592) at baseline, 62,327 (27,097–96,304) at the 3-month follow-up, and 59,518 (34,235–90,046) at the 12-month follow-up.

### 3.5. Alpha-Diversity Findings

**Figure 2:**
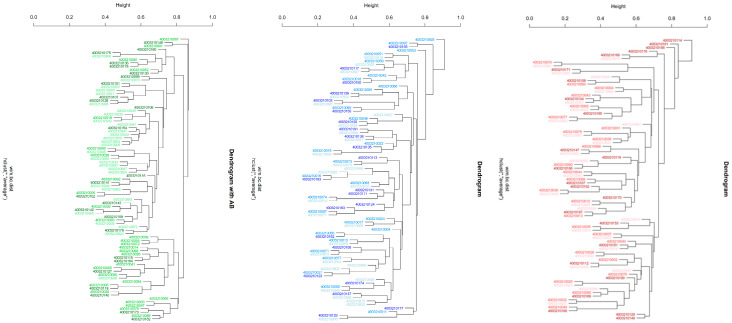
Hierarchical clustering (hclust) of beta-diversity findings across the plaque samples collected from the three study groups. Different shades of the same colors are used for the three collection times. The PT1 group is indicated in green, the PT2 group in blue, and the control group in red shades. The 10-digit numbers across the trees serve as sample identifiers. * describes the average linkage method in hclust.

**Figure 3:**
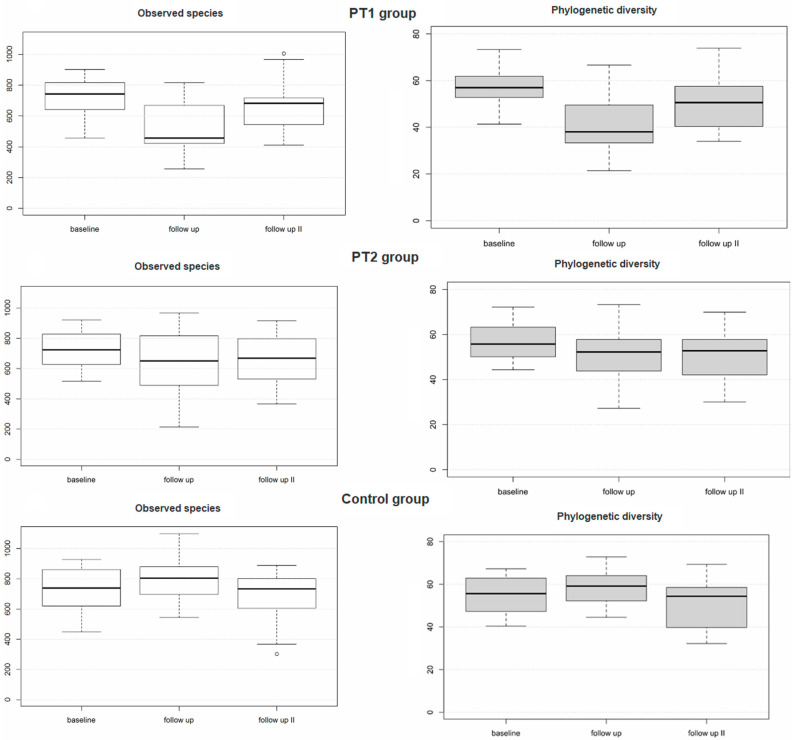
Alpha diversity of the plaque samples (*n* = 228) as evaluated for the three study groups over time based on descriptive statistical data.

The taxonomic ranks detected over all samples are detailed in Figure 2. As shown in Table 3, the PT1 group revealed highly significant differences from baseline to 3 months for Shannon index (*adj. p =* 0.000078), observed species (*adj. p =* 0.000025), and phylogenetic diversity (*adj. p =* 0.000001). In fact, significant changes in the PT1 group were noted for all alpha-diversity metrics between any of the three collection times, except for two non-significant changes from baseline to 12 months for Shannon index (*adj. p =* 0.083) and observed species (*adj. p =* 0.065). The PT2 group revealed no significant changes between the three collection times for Shannon index (*adj. p =* 0.095) or observed species (*adj. p =* 0.057). Phylogenetic diversity did significantly differ from baseline both to 3 months (*adj. p =* 0.005) and to 12 months (*adj. p =* 0.025) within this group (Table 3 and Figure 3). The control group did reveal significant differences between baseline and 3 months for Shannon index (*adj. p* = 0.034) and phylogenetic diversity (*adj. p =* 0.028) but not—between any of the three collection times—for observed species (*adj. p* = 0.094). 

### 3.6. Beta-Diversity Findings

**Figure 4:**
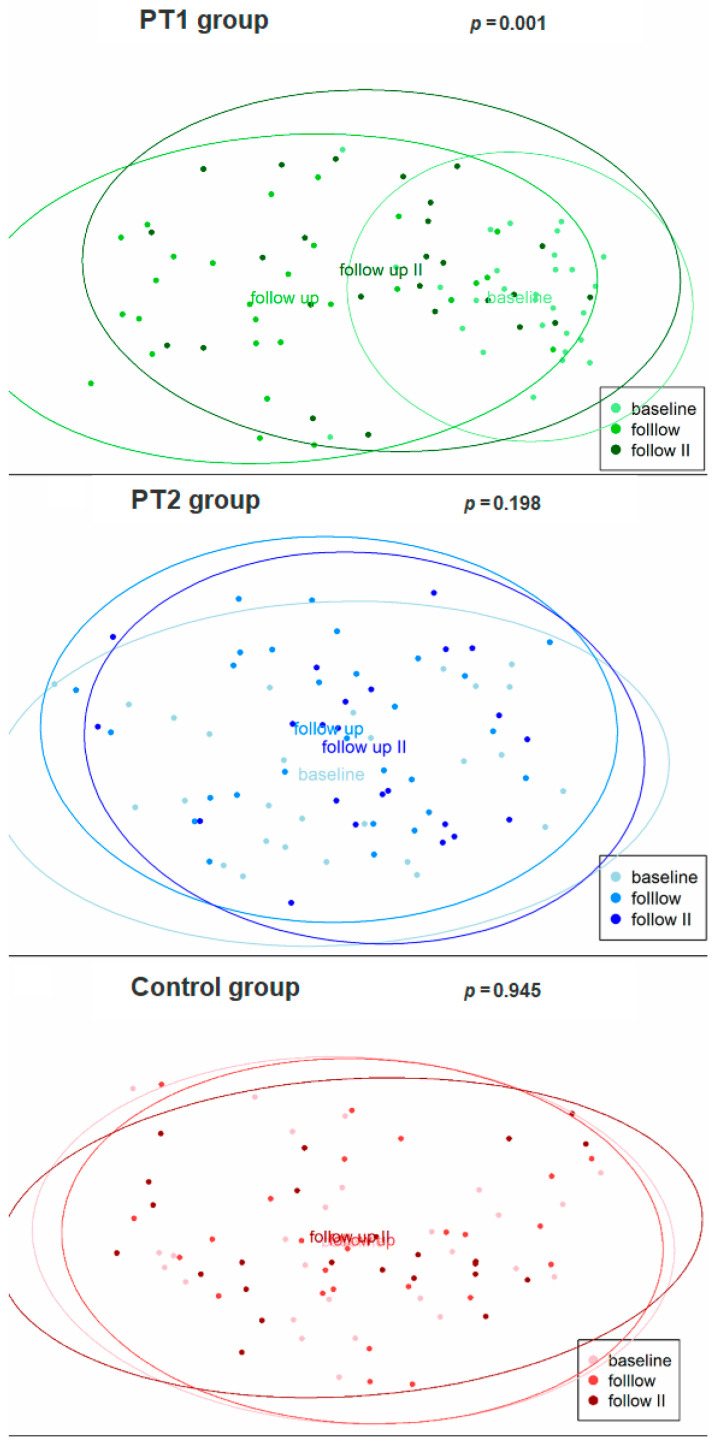
Beta diversity of the plaque samples (*n* = 228) as evaluated for the three study groups over time by principal coordinate analysis. The baseline and follow-up (3 and 12 months) collection times are indicated by lighter to darker shades.

Table 3 also summarizes the results for beta diversity. Significant differences were, both exclusively and consistently, obtained within the PT1 group: from baseline to 3 months (*adj. p =* 0.003) and 12 months (*adj. p =* 0.003), and from 3 to 12 months (*adj. p =* 0.003). This was confirmed by principal coordinate analysis (Figure 4), again disclosing highly significant differences between the three collection times in the PT1 group (*p* = 0.001) but not in the PT2 or control group (*p* = 0.198; 0.945). Hierarchical clustering of all samples, too, revealed significant differences in distance along the tree of samples within the PT1 group only (Figure 2).

### 3.7. Differential Abundances of Taxa Between the Three Timepoints

**Figure 5:**
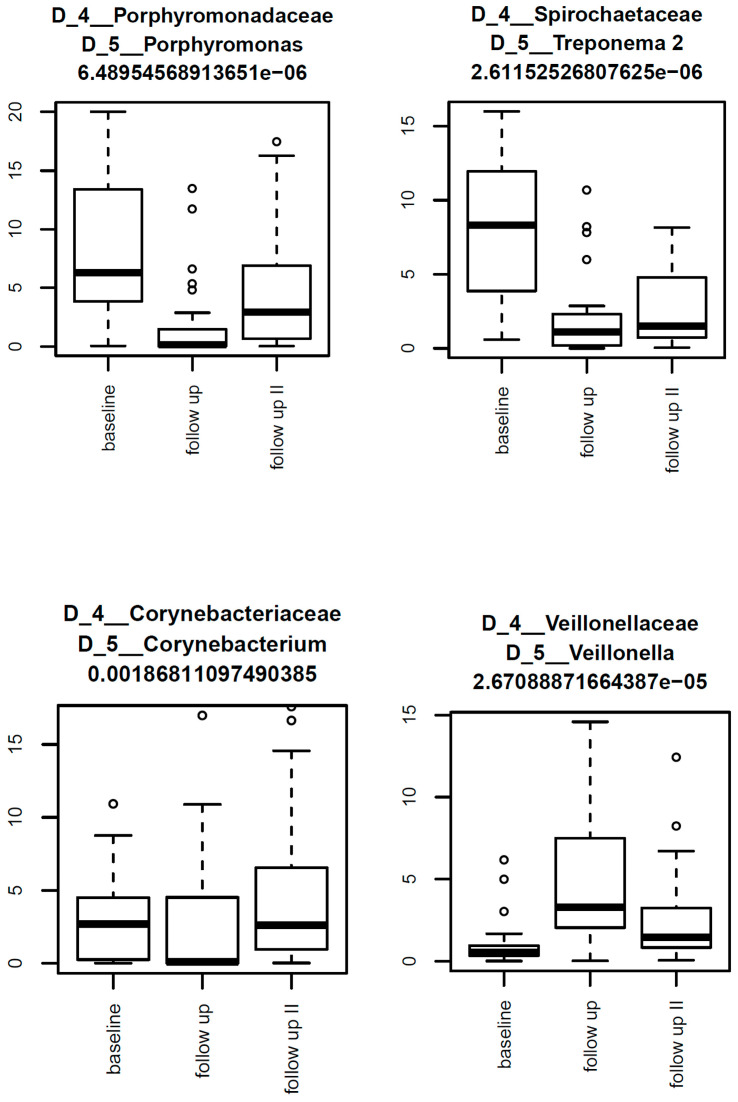
Box-and-whisker plots illustrating the most abundant taxa within the PT1 group over the three collection times. The highest abundances here were seen for *Porphyromonas*, *Treponema 2*, *Corynebacterium*, and *Veillonella*.

Despite this uniform picture of differences in beta diversity over time being limited to the PT1 group, each of the three groups was additionally subjected to LIMMA analysis for differential abundances of taxa between the three collection times. As apparent from Table 4, neither the PT2 nor the control group exhibited any differential abundances. In the PT1 group, significantly different taxa were identified from baseline to 3 months (*n* = 42), from baseline to 12 months (*n* = 11), and from 3 to 12 months (*n* = 9; Table 4 and Figure 5). The precise taxa are broken down in Figure 2 and Figure 5.

## 4. Discussion

To the authors’ knowledge, this is the first randomized controlled study to examine subgingival microbiome changes in different groups of periodontal treatment over 12 months of follow-up, including a control group that did not receive treatment for the first 3 months. The inclusion of such a temporarily untreated control group, which received only supragingival cleaning and oral hygiene instructions, reflects a clinically and ethically acceptable delay, consistent with real-world protocols where hygiene phases may extend over several weeks. The results for microbiome changes over this period demonstrate a beneficial impact of adjunctive systemic antibiotics on the clinical and microbiological outcomes after non-surgical management of stage III/IV periodontitis. This is consistent with reports on subgingival microbial changes by adjunctive systemic antibiotics in stage III/IV periodontitis [13] and on modulated microbial compositions with improved clinical outcomes, particularly in progressing sites, by non-surgical treatment of stage II/III periodontitis [32,33].

### 4.1. PISA Scores and Pocket Depths

Consistent with previous reports [16], this study found non-surgical periodontal treatment with adjunctive systemic antibiotics (PT1 group) to entail more favorable PISA scores and probing depths notably at the 3-month follow-up. Compared to the same treatment without antibiotics (PT2 group), only the differential improvements in PISA scores were significant by 3 months (*p* = 0.001). To put this into perspective, improved PISA scores with antibiotics are due in part to the constituent bleeding score. The PT1-vs.-PT2 results for probing depths were, presumably due to the limited sample size, not significant by 3 months (Table 2). That said, the trends apparent from Table 1 for more shallow pockets (by 7.52 ± 9.71 vs. 2.86 ± 7.19 percentage points [pp]) compared with fewer moderate (by 3.66 ± 8.81 vs. 0.75 ± 5.43 pp) or advanced (by 3.86 ± 5.15 vs. 2.21 ± 4.45 pp) pockets appear conclusive even in the absence of statistical significance (*p* = 0.103/0.310/0.321 by post hoc analysis; *p*-values not shown in Table 2). Compared to the control group not receiving treatment, most changes in the PT1 group were statistically significant over the entire study period (i.e., from baseline to 12 months; Table 2). Yet the finding of no significant changes from 3 to 12 months (Table 2) might indicate a need for continued maintenance therapy, with appointments for supportive periodontal care every 3 months to preserve the clinical gains throughout the first year [34].

This suggested 3-month interval is aligned with the EFP’s risk-based S3-level guidelines and reflects both the early relapse potential of deep pockets and the limited patient adherence seen with longer intervals. While the feasibility of such frequent recall may depend on health system infrastructure and patient motivation, it is considered practical in many specialist or high-risk settings.

### 4.2. Alpha and Beta Diversities

Microbiome analysis of the subgingival plaque samples did reveal distinct patterns of microbial change associated with the three different management protocols. In the PT1 group, the alpha-diversity metrics of Shannon index (species richness and evenness) and Faith’s PD (phylogenetic diversity) had significantly changed over time. Hence major shifts in microbial composition must have occurred specifically as a function of the antibiotics prescribed, given that similar changes were not seen in the PT2 or control group. Likewise, beta-diversity analysis (metrics being Bray–Curtis dissimilarity or weighted UniFrac) revealed significant differences between all three times of sample collection within the PT1 group, compared to no such differences within the PT2 or control group. More confirmation to the same effect came from principal coordinate analysis and hierarchical clustering, all indicating a remarkable impact of systemic antibiotics on the subgingival microbiome (see Figure 2 and Figure 4). These changes may reflect a shift toward a more eubiotic or “health-compatible” microbiota, although such a designation remains operational rather than absolute. Typically, this is inferred from decreases in anaerobic, proteolytic, and inflammation-associated taxa alongside increases in early colonizers or species associated with tissue homeostasis [35,36].

### 4.3. Differential Abundances of Taxa

LIMMA analysis revealed a great many differential abundances of taxa within the PT1 group, including, but not limited to, significant reductions in key periodontal pathogens such as *Porphyromonas*, *Treponema 2*, and *Fretibacterium* between baseline, 3 months, and 12 months. These taxa are strongly implicated in chronic inflammation, immune evasion, and tissue destruction, with *Porphyromonas gingivalis*, in particular, regarded as a keystone pathogen due to its ability to disrupt host-microbial homeostasis [6]. *Treponema* and *Fretibacterium* spp. are known to dominate in deep pockets and contribute to proteolytic activity and epithelial barrier breakdown, reinforcing the clinical relevance of their suppression. Reducing these could, therefore, as evidenced by the PT1 group, greatly improve the periodontal status of patients. Surprisingly, albeit consistent with the aforementioned microbiological findings, neither the PT2 nor the control group exhibited any differential abundances, once again confirming the superiority of the combined approach as in the PT1 group.

### 4.4. Limitations and Future Directions

This study did not include, as a benchmark for ‘health-oriented’ microbiome changes, a control group without periodontal disease. Also, the reported cohort with stage III/IV periodontitis was both relatively small and characterized by a specific co-morbidity (peripheral artery disease). This vascular condition may have influenced both host immune responses and microbiome dynamics, potentially limiting the generalizability of the findings to otherwise healthy periodontitis patients. More studies with larger case numbers and more diverse patients will be required to elucidate long-term implications for subgingival microbiota and clinical outcomes. In addition, other antimicrobial agents should be explored in an effort to optimize the protocols available for combined treatment of periodontal disease. In future research, adverse events, resistome dynamics, and long-term compositional shifts related to systemic antibiotic use should be explicitly monitored. Recent studies suggest that oral antibiotic use can enrich resistance genes and promote horizontal gene transfer within oral microbial communities, even after short-term exposure [37,38]. While the clinical benefits observed in our study are encouraging, the potential drawbacks—including dysbiosis, gastrointestinal side effects, and especially antimicrobial resistance—must not be underestimated. These issues underscore the importance of using systemic antibiotics judiciously and only in accordance with established clinical guidelines such as the EFP’s S3-level recommendations, which advocate for a restricted and evidence-based approach.

### 4.5. Implications for Periodontal Therapy

Our implied finding that diminishing PISA scores were associated with ‘health-oriented’ microbial shifts is consistent with other studies [35,36,39]. The term “health-oriented” here refers to compositional changes away from anaerobic proteolytic consortia toward more stable, less inflammatory communities. We reiterate observing that the clinical effects decreased from 3 to 12 months, suggesting that continued maintenance or repeated supportive therapy may be indicated to ensure long-term periodontal health. Overall, however, our results are consistent with reports on better efficacy of scaling and root planing with systemic antibiotics [13,40,41].

### 4.6. Conclusions

Any decision to prescribe antibiotics should be carefully considered, given their potential for dysbiosis and resistance. Indeed, resistant bacteria can cause severe health issues and even death [42]. Gastrointestinal side effects are also a frequent concern. In light of these risks, systemic antibiotics should not be used routinely in periodontal therapy but rather reserved for cases where the anticipated benefit outweighs the risks. This principle aligns with the EFP’s current S3-level clinical practice guidelines, which call for a strict indication and careful patient selection. Taking a scrupulous risk–benefit assessment for granted and bearing in mind the specific patient characteristics of this study, our microbiological and clinical data offer a proof of principle that non-surgical periodontal therapy can reduce the inflammatory burden more effectively when combined with adjunctive systemic antibiotics, in carefully selected cases.

## Figures and Tables

**Table 3:** Comparison of the microbiota found in all time-related biofilm samples (*n* = 228) collected across the three study groups at baseline and during the follow-up visits.

Group					Alpha Diversity			Beta Diversity
	Time	vs.	Time		Shannon Index	Observed Species	Phylogenetic Diversity	
PT1 group	Baseline	vs.	3 months	*adj. p*	0.000078 **	0.000025 **	0.000001 **	0.003 *
	Baseline	vs.	12 months	*adj. p*	0.083	0.065	0.007 *	0.003 *
	3 months	vs.	12 months	*adj. p*	0.007 *	0.011 *	0.005 *	0.003 *
PT2 group	Baseline	vs.	3 months	*adj. p*	↓	↓	0.005 *	↓
	Baseline	vs.	12 months	*adj. p*	0.095	0.057	0.025 *	0.228
	3 months	vs.	12 months	*adj. p*	↑	↑	0.706	↑
Control group	Baseline	vs.	3 months	*adj. p*	0.034 *	↓	0.028 *	↓
	Baseline	vs.	12 months	*adj. p*	0.975	0.094	0.321	0.929
	3 months	vs.	12 months	*adj. p*	0.275	↑	0.275	↑

Asterisks next to “*adj. p*” (*=adjusted p*) values indicate significant (*) or highly significant (**) differences. Arrows pointing to *p*-values indicate that the difference contained within the three comparisons was not significant. ‘Shannon index’ is a metric of species richness/evenness, ‘observed species’ a simple metric of richness, and the metric of ‘phylogenetic diversity’ is based on Faith’s PD. Beta diversity was assessed by the ‘adonis’ function as Bray–Curtis dissimilarity (‘weighted UniFrac’ for the significant PT1 results).

**Table 4:** LIMMA analysis for differentially abundant genera in all biofilm samples (*n* = 228).

				PT1 Group			PT2 Group			Control Group		
Time	vs.	Time		C − 1	C0	C1	C − 1	C0	C1	C − 1	C0	C1
Baseline	vs.	3 months	*n* of taxa	31 *	119	11 *	0	159	0	0	154	0
Baseline	vs.	12 months	*n* of taxa	8 *	150	3 *	0	159	0	0	154	0
3 months	vs.	12 months	*n* of taxa	1 *	152	8 *	0	159	0	0	154	0

C = contrast: While C0 indicates no changes in taxa, any non-zero values for C1 and C − 1, marked by asterisks (*), indicate significant changes between times. LIMMA: linear models for microarray data.

## Data Availability

All FastQ raw data can be accessed via SRA accession number PRJEB82494 at the European Nucleotide Archive (ENA).

## Supplementary Materials

The following supporting information can be downloaded at: https://www.mdpi.com/article/10.3390/diagnostics15162012/s1. The Supporting Information to this article comprises three Excel files. Supplementary File S1: Inclusion and exclusion criteria. Supplementary Figure S1: Hierarchical clustering of all samples in the three treatment groups. Supplementary Figure S2: Box plots of differentially abundant genera found by limma anlysis in the AB group. Supplementary Table S1: Taxonomic ranks detected over all plaque samples from the three study groups. The file includes spreadsheets for phyla, classes, orders, families, and genera. The main phyla were Bacteroidetes, Fusobacteria and Firmicutes; the main genera were *Fusobacterium*, *Porphyromonas*, *Streptococcus*, *Treponema 2*, and *Leptotrichia.* Supplementary Table S2: Data of alterations in differentially abundant taxa, based on the plaque samples from the PT1 group. The file includes spreadsheets for alterations from baseline to 3 months, baseline to 12 months, and 3 months to 12 months. At 3 months, the highest abundances over all PT1 samples were seen for *Porphyromonas*, *Treponema 2*, *Corynebacterium* and *Veillonella*, while the three most abundant genera at baseline had been *Porphyromonas*, *Treponema 2*, and *Fretibacterium*. The highest fold changes were found for an uncultured bacterium of Lactobacillales, *Neisseria*, *Granulicatella*, an uncultured genus of Porphyromonadaceae, *Desulfobulbus*, and unknown genera of *Saccharibacteria*. Changes from baseline to 12 months were seen for 11 taxa, the most abundant across all samples being *Treponema 2*, *Fretibacterium*, and *Lautropia*. From 3 months to 12 months, 9 taxa were found to remain statistically significant, including *Porphyromonas*, *Corynebacterium*, and *Tannerella*. Supplementary Table S3: Dataset of a ‘random forest feature selection analysis’ of significantly altered taxa, based on the plaque samples from the PT1 group. The differences observed in this group were mainly due to *Shuttleworthia*, *Phocaeicola*, *Lactobacillus*, and *Stomatobaculum.*
