# Supplementary figures and images for: Impact of Antibiotics on the Subgingival Microbiome in Advanced Periodontitis: Secondary Analysis of a Randomized Controlled Trial
Source: Diagnostics (Basel). 2025 Aug 11;15(16):2012. doi: 10.3390/diagnostics15162012 (PMC12385641; doi:10.3390/diagnostics15162012)

## Slide 1
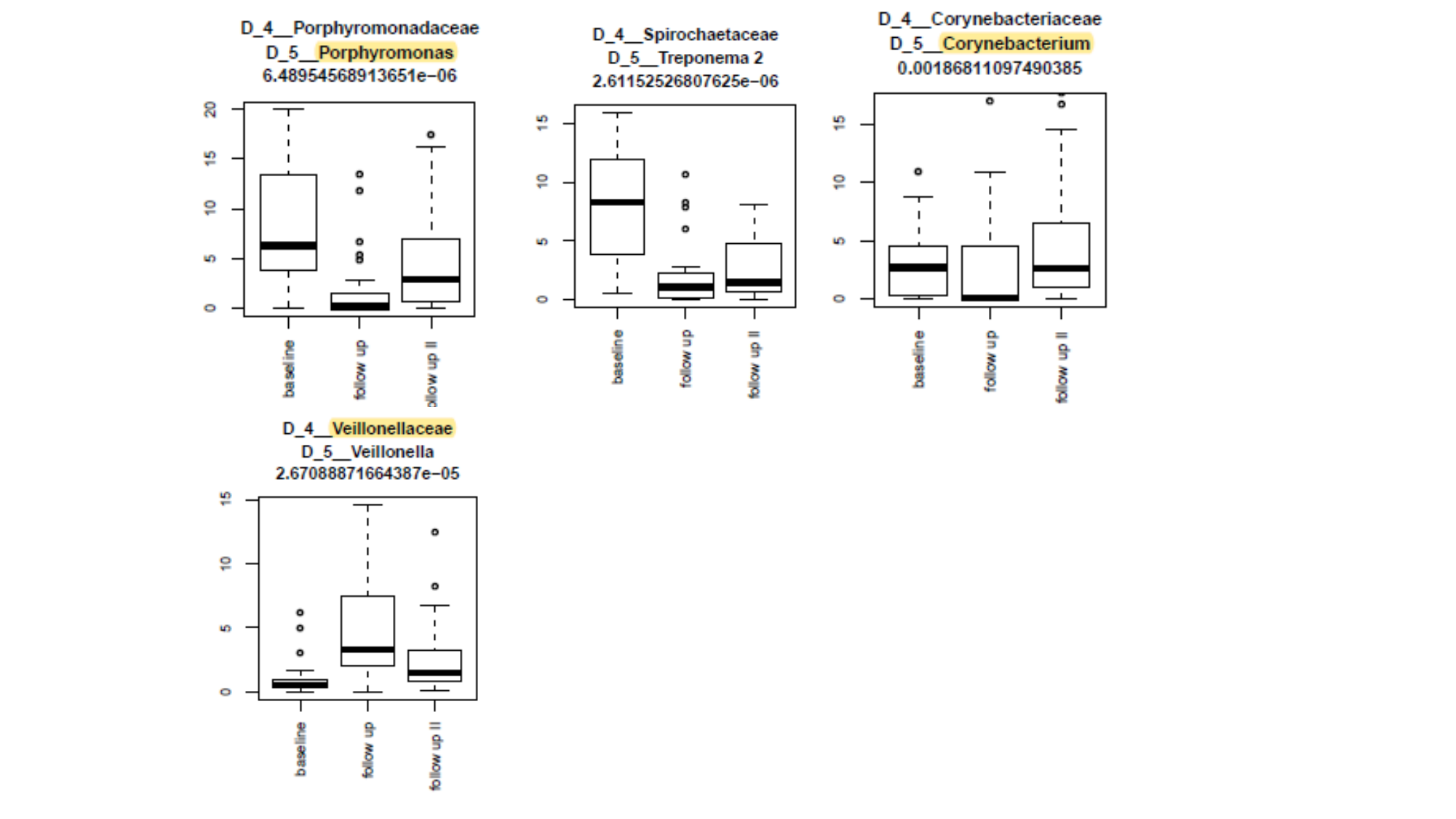

Supplement: Supplementary file 1 [file diagnostics-15-02012-s001.zip › Suppl. Fig.S2.pptx]

## Slide 1
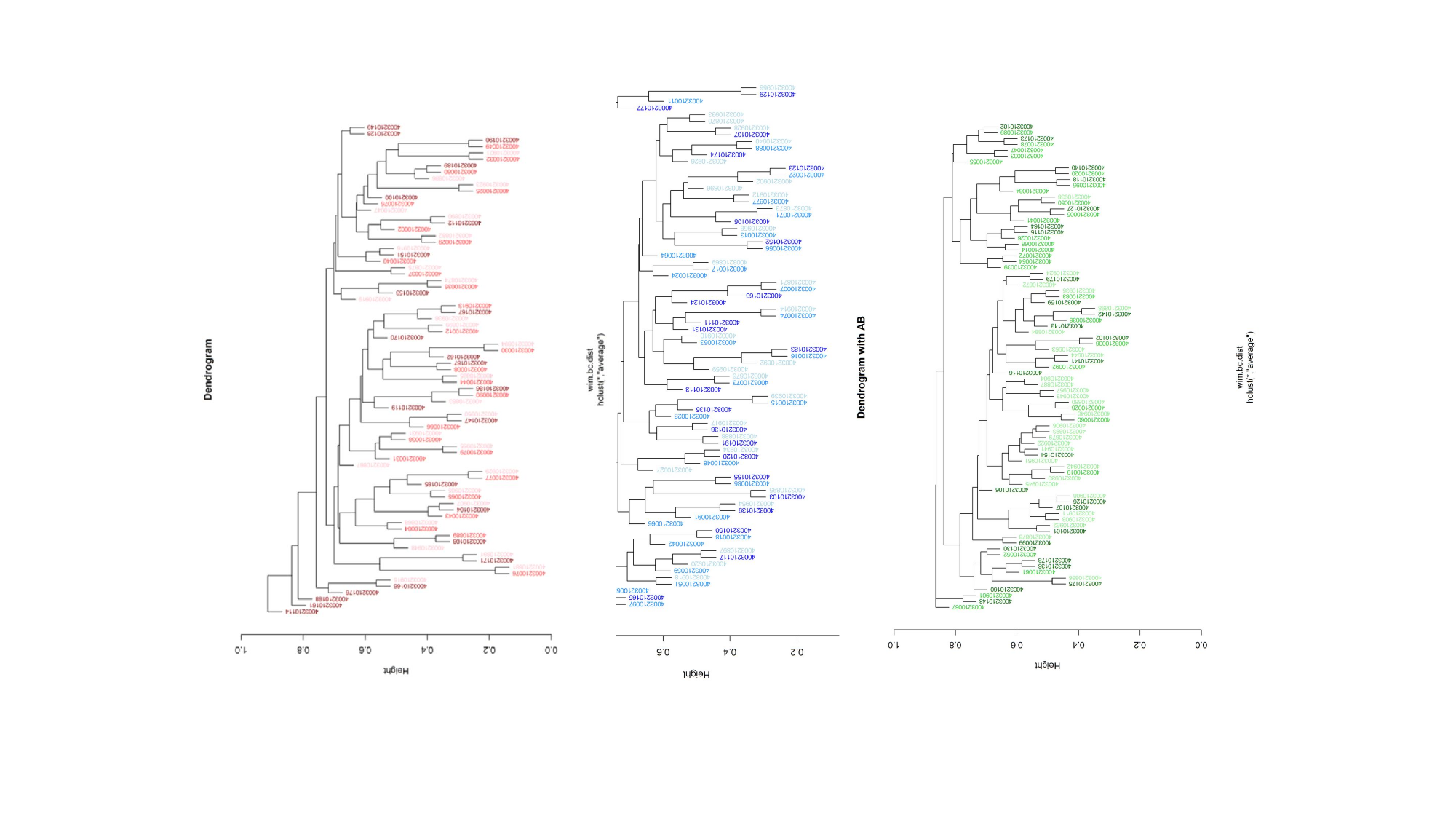

Supplement: Supplementary file 1 [file diagnostics-15-02012-s001.zip › Suppl.Fig.S1.pptx]
